# The swing performance Index: Developing a single-score index of golf swing rotational biomechanics quantified with 3D kinematics

**DOI:** 10.3389/fspor.2022.986281

**Published:** 2022-12-23

**Authors:** Joanne Y. Zhou, Alexander Richards, Kornel Schadl, Amy Ladd, Jessica Rose

**Affiliations:** ^1^ Department of Orthopaedic Surgery, Stanford University, Stanford, CA, United States; ^2^ Motion & Gait Analysis Lab, Lucile Packard Children's Hospital, Palo Alto, CA, United States

**Keywords:** golf swing technique, rotational parameters, wearable sensors, performance index, biomechanics

## Abstract

**Introduction:**

Golf swing generates power through coordinated rotations of the pelvis and upper torso, which are highly consistent among professionals. Currently, golf performance is graded on handicap, length-of-shot, and clubhead-speed-at-impact. No performance indices are grading the technique of pelvic and torso rotations. As an initial step toward developing a performance index, we collected kinematic metrics of swing rotational biomechanics and hypothesized that a set of these metrics could differentiate between amateur and pro players. The aim of this study was to develop a single-score index of rotational biomechanics based on metrics that are consistent among pros and could be derived in the future using inertial measurement units (IMU).

**Methods:**

Golf swing rotational biomechanics was analyzed using 3D kinematics on eleven professional (age 31.0 ± 5.9 years) and five amateur (age 28.4 ± 6.9 years) golfers. Nine kinematic metrics known to be consistent among professionals and could be obtained using IMUs were selected as candidate variables. Oversampling was used to account for dataset imbalances. All combinations, up to three metrics, were tested for suitability for factor analysis using Kaiser-Meyer-Olkin tests. Principal component analysis was performed, and the logarithm of Euclidean distance of principal components between golf swings and the average pro vector was used to classify pro vs. amateur golf swings employing logistic regression and leave-one-out cross-validation. The area under the receiver operating characteristic curve was used to determine the optimal set of kinematic metrics.

**Results:**

A single-score index calculated using peak pelvic rotational velocity pre-impact, pelvic rotational velocity at impact, and peak upper torso rotational velocity post-impact demonstrated strong predictive performance to differentiate pro (mean ± SD:100 ± 10) vs. amateur (mean ± SD:82 ± 4) golfers with an AUC of 0.97 and a standardized mean difference of 2.12.

**Discussion:**

In this initial analysis, an index derived from peak pelvic rotational velocity pre-impact, pelvic rotational velocity at impact, and peak upper torso rotational velocity post-impact demonstrated strong predictive performance to differentiate pro from amateur golfers. Swing Performance Index was developed using a limited sample size; future research is needed to confirm results. The Swing Performance Index aims to provide quantified feedback on swing technique to improve performance, expedite training, and prevent injuries.

## Introduction

Golf is a popular sport with approximately 37 million participants in the United States (1). The modern golf swing is a complex and asymmetrical movement that harnesses body rotational mechanics to optimize driving distance and direction. A successful swing is challenging to achieve, and poor swing dynamics can lead to injury. Performance indices can efficiently provide evaluation, comparison, and outcome assessment, summarizing the quantitative data captured by motion analysis on athletic technique.

With each golf swing lasting just one second on average, it is important to identify swing deficits to provide real-time feedback and quantify adjustments in technique for performance optimization and injury prevention. Professional golf swings involve motions that are smooth, cadenced, and efficient. Peak rotational velocity must be translated through the club while maintaining control and precision. Biomechanical factors influencing golf swing power generation have previously been characterized as pro swing benchmark curves to better understand the differences between amateur and professional golfers (2). Benchmark curves of angle of rotation and rotational velocities of the pelvis, upper torso, and X-prime (defined as the rate of change of the relative angular position between the pelvis and upper torso along the transverse plane) have previously been established throughout the pro golf swing, shown to be consistent in pros, and significantly different between amateurs and professionals (3). Professional golf instructors and several studies (2, 3) have emphasized the importance of absolute and relative pelvic and upper-torso rotation, pelvic rotation, translation, and free moment of force that translates from golfer to ball during the golf swing. Cheetham et al. used wearable sensors and 3D motion analysis to quantify segments of the golf swing and found that the timing sequence of rotational velocity peaks consistently differentiated pro from amateur golfers. Their findings support the proximal to distal sequencing theory first introduced by Cochran and Stobbs in 1968 (4) in that pros have maximum rotational velocity first at pelvis, then thorax, then arm and finally club. In amateurs, they found that the mean arm peak time is before the mean thorax peak time which suggests that amateurs tend to use their arms earlier in the downswing than the pros and had greater variability in their swing sequencing and timing (5). Other studies have also emphasized the importance of proximal to distal body segment sequencing (6) and importance of X-factor timing, specifically that a greater increase in the X-factor early in the downswing correlates with pro golf swing (7). We sought to measure the rotational parameters of the golf swing and elucidate whether single parameters or a combination of parameters in a performance index could differentiate individual golf swings at pro or amateur levels.

In elite athletics, there are ongoing attempts to measure players' games. Various player statistics are used, such as ranking points, money earned, top finishes, and scores. Studies have used earnings (8–12) or scoring average (8, 9, 12, 13) as the dependent variable to examine the relative importance of various parts of the game, such as driving accuracy, greens in regulation, sand saves, and putts per round on athlete performance. Partial scores gather statistics of player history round-by-round, hole-by-hole, or shot-by-shot (14, 15). However, to our knowledge, no index currently summarizes kinematic data from a golf swing into a single score to grade performance.

Performance indices can provide a single score of golf swing technique for evaluation, comparison, and outcome assessment, that summarizes the comprehensive kinematic and temporal-spatial data captured by motion analysis. Several studies have shown that IMUs can be reliably used to identify the various segments of the golf swing (16, 17). It has also been shown that data derived from IMUs have the potential to classify swing technique as proper or improper, where improper swing has a higher likelihood to cause injury (18). Finally, real-time IMU-based analysis of the wrist angle uncocking motion shows the coaching potential of wearable sensors (19). Single-score indices are widely used in gait evaluation to assess walking patterns and are useful in diagnosis and outcome assessment (20–22). To our knowledge, no index has been created that aggregates kinematic measures of the golf swing to grade swing.

Current measures of the golf swing rely on handicap, Clubhead Speed at Impact (CSI), and driving distance. However, none provide direct feedback on rotational technique. Trunk rotation assessed with the upper torso and pelvic rotational velocities, as well as upper torso and pelvic obliquities, are readily measured with kinematic analyses, and deviation from pro swings can provide useful feedback on training progress.

Golf-related injuries commonly occur in the lower back (incidence 15%–36%), shoulders (incidence 6%–10%), wrists (incidence 13%–36%), and elbows (incidence 7%–50%) (23–36). Injury occurs from overuse, primarily in professionals, or trauma and improper swing biomechanics. Improper rotational biomechanics have been shown to increase the torque, shear, and lateral-bending forces experienced by the lumbar spine in professional golfers (37). Another goal of developing a single-score index is to better define optimal swing biomechanical parameters to assist trainees in avoiding motions that may be extreme and ultimately lead to injury.

**Figure 1:**
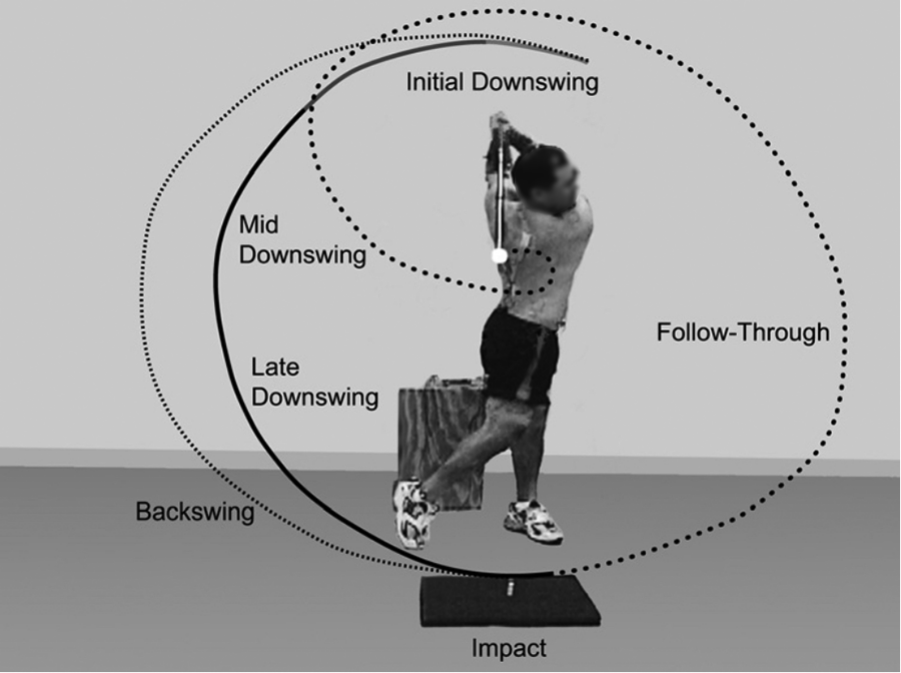
Demonstration of the primary phases of the golf swing as determined by clubhead position: address, backswing, downswing, impact, and follow-through (46).

This research calculates an index of golf swing rotational biomechanics assessed with 3D kinematic metrics. We sought to create a single-score index, the Swing Performance Index (SPI), that could quantify how much an individual golf swing differs from typical pro golf swings using biomechanical parameters that had been found to be consistent in pro golf swings and could be derived using inertial measurement units (2, 3). The SPI seeks to standardize pro golf swings to a score of 100 ± 10 (mean ± SD), with lower values being indicative of suboptimal upper torso and pelvic rotational biomechanics. This index builds upon prior pro swing benchmark analyses throughout the swing phases (Figure 1) (2, 3, 4). In creating the golf SPI, we considered the inclusion of kinematic metrics thought to be technically relevant, reported in the literature, and those that demonstrated statistical significance in differentiating amateurs from pros in our data set.

We hypothesized that the golf SPI will differentiate between amateur and pro golfers.

## Methods

### Participant recruitment and data collection

Data were included from eleven pros and five amateurs, recorded at the Motion & Gait Analysis Lab at Lucile Packard Children's Hospital at Stanford, CA (2, 3). This study was approved by the Stanford University Institutional Review Board (protocol ID# 11910). Inclusion criteria were amateur or professional male golfers with no musculoskeletal injury that might impact their golf swing. Professional golfers were members of the Professional Golfers' Association (38). To include a range of skill levels for comparison, amateur golfers included one with a low handicap (handicap = 4), one with a medium handicap (handicap = 15), one with a high handicap (handicap = 30), and two novices who did not play regularly (handicap unknown). All participants provided informed consent.

For each subject, a simplified marker set using reflective markers with a diameter of one centimeter was used to evaluate rotational velocities of the pelvis and upper torso. Reflective markers were placed on the anterior superior iliac spines (ASIS) bilaterally to evaluate pelvic motion, and on the acromia bilaterally to evaluate upper torso motion. The orientation of the pelvis and upper torso was determined by subtracting the 3D vectors of the left and right ASIS, and the left and right acromion marker positions, respectively. Rotational velocities were obtained by calculating the rate of change of the pelvic and upper torso orientation along the transverse plane using Python and NumPy (39, 40). A marker was also placed at the distal end of the club shaft at 5 cm from the center of the clubface, and a plastic practice ball was wrapped in reflective tape to identify ball contact and Clubhead Speed at Impact (CSI). An 8-camera motion capture system (Motion Analysis Corporation, Rohnert Park, CA, USA) was used to capture marker positions with a sampling rate of 240 Hz.

All participants were given an opportunity to warm up in the testing area before the swings were recorded. Golfers then performed three hard swings using their own five iron from which the two best swings with minimal marker dropout were analyzed. For pro golfers, both of these two swings were processed. Amateur golfers had less experience controlling their golf swing speed; therefore, only the swing with the highest CSI was processed.

### Definitions of the golf swing cycle and kinematic metrics

Each golf swing was analyzed based on the phases of the golf swing cycle (Figure 1). The start of the backswing was determined by the first point in time with a substantial increase in vertical displacement of the clubhead. Based on previous studies and confirmed through visualization of individual golf swings using the motion capture software, a vertical clubhead speed greater than 0.2 m/s after address was found to consistently identify the beginning of each golf swing (2, 3). Impact was defined as the time point immediately preceding the initial increase in ball velocity. The end of follow-through was defined as the first local minimum in vertical clubhead displacement following impact.

Orientation of the upper torso and the pelvis were calculated independently and were obtained by connecting a virtual segment across the acromion markers for the upper torso, and the ASIS markers for the pelvis. Rotational velocities were determined by calculating the rate of change of the orientation of the virtual segments along the transversal plane, measured in degrees per second. X-prime was defined as the difference between the pelvic and upper torso rotational velocity at each time point throughout the swing (2).

Clubhead speed was calculated from the change in position of the marker attached to the distal end of the shaft between impact and the immediate next time point. Since the marker on the distal end of the shaft was located 5 cm from the center of the clubface, we approximated clubhead speed by fitting a circle to the trajectory of the distal shaft in the 50 time points immediately preceding and after impact and adding 5 cm to the radius of the resulting circle.

### Data analysis

**Figure 2:**
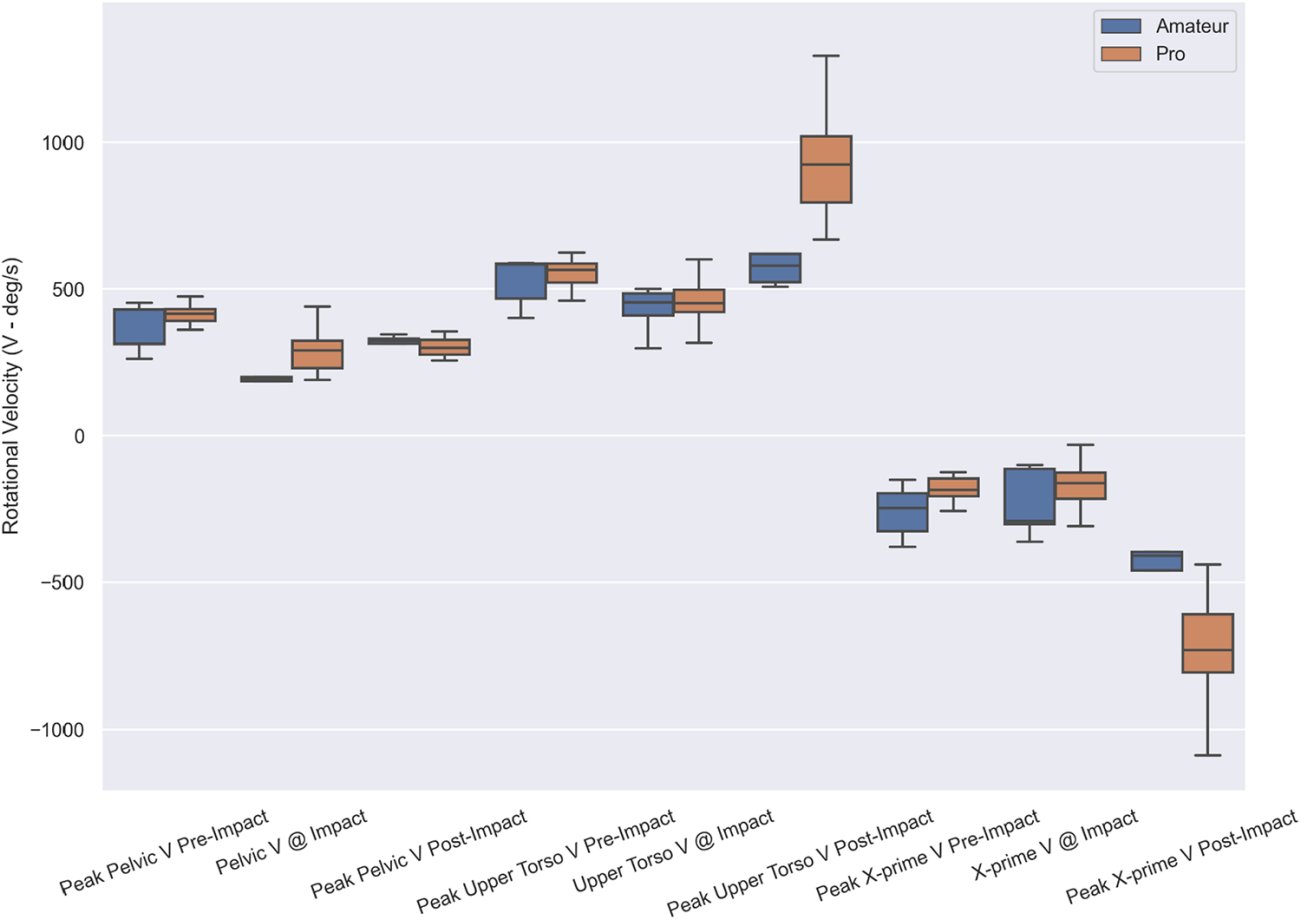
Box plot of individual extracted kinematic metrics of pro vs. amateur golf swings showing the median, minimum, maximum, 25, and 75 percentiles.

Nine candidate kinematic metrics (Figure 2) were considered for the construction of the golf SPI, including peak rotational velocity pre-impact, at impact, and post-impact of the pelvis, upper torso, and X-prime. Before performing the index computation, the kinematic metrics were normalized to zero mean and unit variance using the professional group's distribution.

All combinations of up to three kinematic metrics were tested using the Kaiser-Meyer-Olkin (KMO) test to determine if the set of variables is suitable for factor analysis (41). KMO values below 0.6 were considered inadequate and were excluded from further analysis. For each combination suitable for factor analysis, a distinct copy of the original dataset containing only the kinematic metrics determined by the combination was used for further processing.

To lower potential bias and better capture differences between the two groups, the dataset of the amateur group was oversampled using the Synthetic Minority Over-sampling Technique (42) to account for imbalance in data containing more than twice the number of participants in the professional over the amateur group. A sample from the amateur group and its nearest neighbor in the feature space was chosen, and a randomly selected point between the two vectors was added to the dataset. This process was repeated until the number of samples in the two groups were equal.

To decouple interrelated parameters into independent components, for each combination, a principal component analysis (PCA) was performed. Principal components of each swing were calculated by applying the PCA transformation to the dataset, using PCA parameters determined by the subset of kinematic metrics of the professional group.

For generating the SPI, the logarithm of the Euclidean distance of the principal components between the swings and the average pro swing vector was calculated. A candidate SPI was calculated by scaling these values so that the mean and standard deviation of the professional golf swings were 100 and 10, respectively.

Each candidate SPI were evaluated using logistic regression with leave-one-out cross-validation to assess their predictive performance in classifying pro vs. amateur swings. The area under the receiver operating characteristic curve (AUC) of the cross-validated model was used to determine the optimal set of kinematic metrics to be used.

## Results

Data from eleven pro and five amateur golfers were included in the calculation of the golf SPI (Table 1). The mean age of pro golfers was 31.0 ± 5.9 years, and the mean age of amateur golfers was 28.4 ± 6.9 years (Table 1).

**Table 1:** Participant characteristics.

	Professional (*n* = 11)	Amateur (*n* = 5)
Height (m)	1.83 ± 0.07	1.78 ± 0.03
Mass (kg)	85.9 ± 11.5	77.3 ± 8.9
Age (year)	31.0 ± 5.9	28.4 ± 6.9

Values are presented as mean ± standard deviation.

Nine candidate variables were tested, including peak pelvic rotational velocity pre-impact, pelvic rotational velocity at impact, peak pelvic rotational velocity post-impact, peak upper torso rotational velocity pre-impact, upper torso rotational velocity at impact, peak upper torso rotational velocity post-impact, peak X-prime velocity pre-impact, X-prime velocity at impact, and peak X-prime velocity post-impact (Table 2, Figure 2).

**Table 2:** Peak rotational velocities (deg/s) of the professional golf swing.

	Upper Torso Rotational Velocity		Pelvis Rotational Velocity		X-prime	
	Mean ± SD	CV	Mean ± SD	CV	Mean ± SD	CV
Down-swing (deg/s)	551.7 ± 47.6	0.086	415.2 ± 32.9	0.079	−183.4 ± 41.4	−0.23
Impact (deg/s)	458.5 ± 73.0	0.159	288.8 ± 70.9	0.245	−170.3 ± 63.0	−0.37
Follow-through (deg/s)	929.2 ± 185.1	0.199	309.8 ± 42.1	0.136	−729.4 ± 160.8	−0.22

SD, standard deviation; CV, coefficient of variation. X-prime: the difference between upper torso rotational velocity and pelvic rotational velocity (the relative orientation between the segments of the bilateral acromia and bilateral ASIS along the transverse axis).

**Figure 3:**
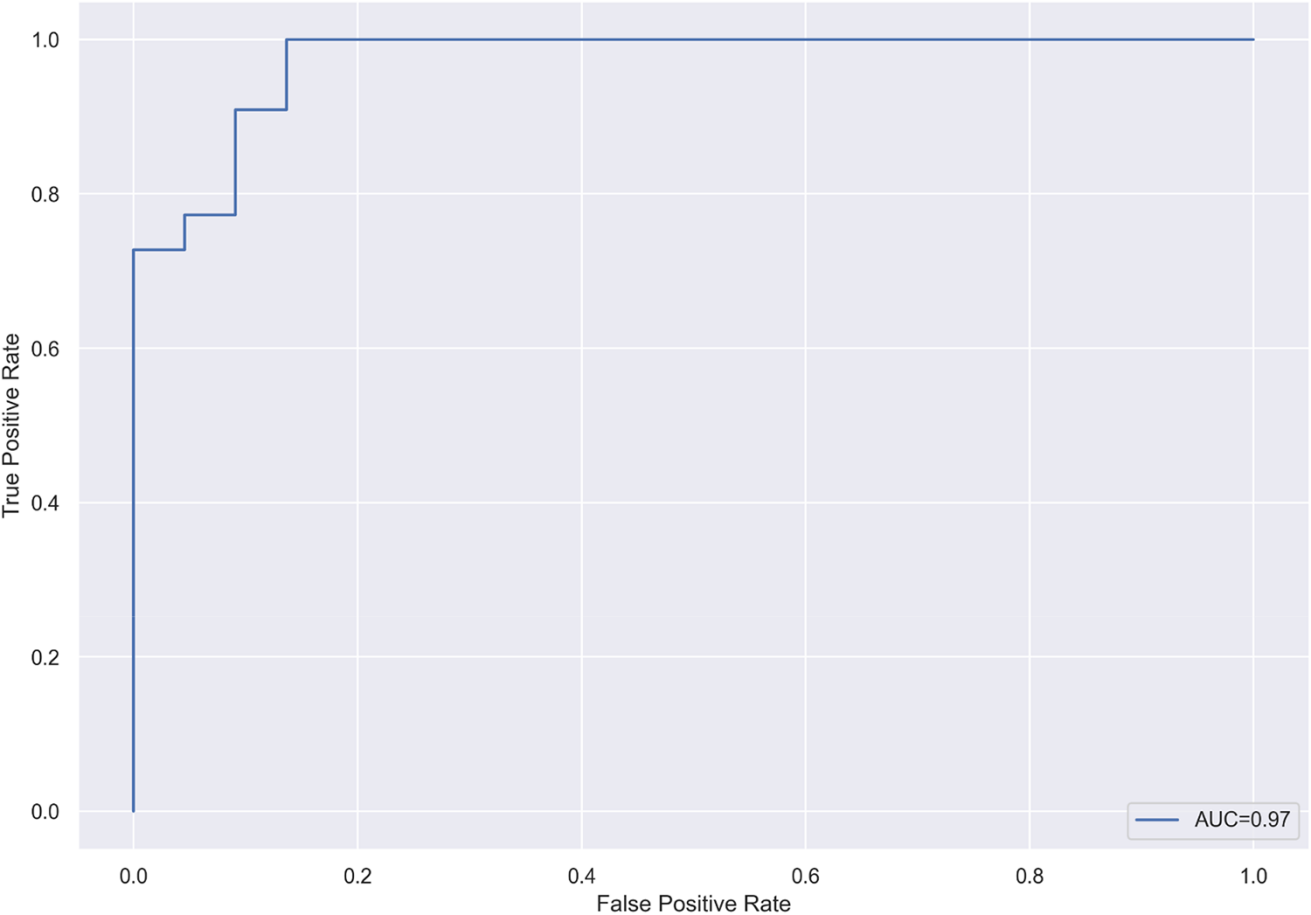
The receiver operating characteristic curve of the cross-validated logistic regression model, differentiating between pro and amateur players, using the swing performance Index based on an optimal set of three kinematic metrics.

A golf SPI calculated using the peak pelvic rotational velocity pre-impact, pelvic rotational velocity at impact, and peak upper torso rotational velocity post-impact demonstrated an optimal set of variables with AUC = 0.97 when pro and amateur swings were classified using logistic regression with leave-one-out cross-validation (Figure 3).

Measurement system analysis values of the KMO test for sampling adequacy were 0.76 for peak pelvic rotational velocity pre-impact, 0.60 for pelvic rotational velocity at impact, 0.57 for peak upper torso rotational velocity post-impact, with an overall KMO value of 0.62 that meets the inclusion criteria (>0.6) for factorability (Table 3).

**Table 3:** Kaiser-Meyer-Olkin (KMO) test for determining suitability for factor analysis of variables selected for inclusion in the swing performance Index.

Variable	KMO
Peak pelvic rotational velocity pre-impact	0.76
Pelvic rotational velocity at impact	0.60
Peak upper torso rotational velocity post-impact	0.57
**Overall**	**0** **.** **62**

**Figure 4:**
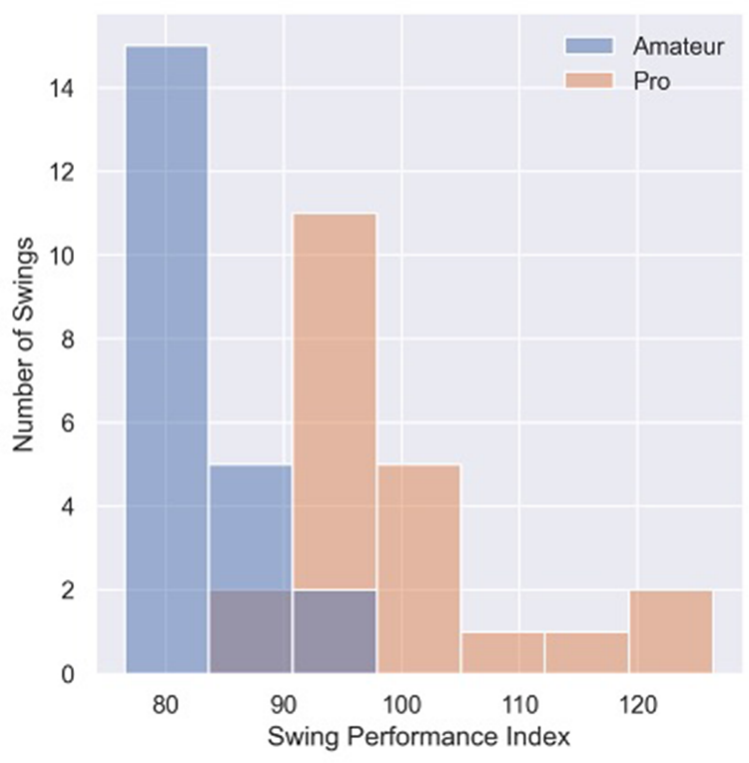
Distribution of swing performance Index for individual swings of pro and amateur golfers.

The mean SPI of pro golfers was 100 ± 10, and the mean SPI of amateur golfers was 82 ± 4 (Table 4 and Figure 4). The standardized mean difference between the two groups was 2.12. The mean SPI of the amateur oversampled dataset was 83 ± 4 (Table 5).

**Table 4:** Mean values of clubhead speed at impact (CSI) and swing performance Index (SPI) for individual pro and amateur participants.

Participant Group	Clubhead Speed at Impact (m/s)	Swing Performance Index
Pro 1	32.85	91
Pro 2	26.20	98
Pro 3	33.89	97
Pro 4	27.66	97
Pro 5	39.57	98
Pro 6	38.67	93
Pro 7	31.15	93
Pro 8	34.27	101
Pro 9	29.12	113
Pro 10	37.13	97
Pro 11	34.14	122
**Pro mean ± SD**	**33.15 ± 5.91**	**100 ± 10**
Amateur 1	30.20	84
Amateur 2	25.20	77
Amateur 3	29.30	80
Amateur 4	34.20	78
Amateur 5	34.00	88
**Amateur mean ± SD**	**30.58 ± 3.33**	**82 ± 4**

**Table 5:** Principal components (PC) and swing performance Index (SPI) of individual swing trials. For amateurs, the oversampled data and derived Swing Performance Index are shown.

Participant Group	1st principal component	2nd principal component	3rd principal component	Swing Performance Index
Pro 1	2.16	−0.47	−0.49	92
Pro 1	2.36	−0.65	0.16	90
Pro 2	0.22	−1.47	−0.80	97
Pro 2	0.23	−1.42	−0.54	98
Pro 3	0.98	1.02	0.25	99
Pro 3	1.27	0.78	1.12	95
Pro 4	−1.22	−0.60	−0.68	99
Pro 4	−1.24	−0.59	−1.12	96
Pro 5	−1.09	−0.72	0.44	100
Pro 5	−0.64	−1.37	1.11	95
Pro 6	−0.16	1.68	0.13	97
Pro 6	0.10	2.50	−0.30	90
Pro 7	−0.52	−0.02	2.00	93
Pro 7	−0.52	−0.21	2.09	93
Pro 8	0.82	−0.09	−1.58	96
Pro 8	−0.64	0.07	−0.74	106
Pro 9	−0.56	0.61	1.02	101
Pro 9	−0.18	−0.17	0.17	126
Pro 10	−0.96	0.87	−0.92	98
Pro 10	−0.72	0.94	−1.38	95
Pro 11	0.21	−0.19	0.05	126
Pro 11	0.09	−0.48	0.00	118
			**Pro mean ± SD**	**100 ± 10**
Amateur	−2.61	−0.36	−2.48	84
Amateur	−3.14	−1.66	−4.28	77
Amateur	−1.20	−2.19	−3.84	80
Amateur	−1.27	2.13	4.37	78
Amateur	−1.10	2.09	1.65	88
Amateur	−1.64	1.23	0.20	93
Amateur	−2.18	0.35	−1.29	90
Amateur	−1.90	−2.00	−4.00	79
Amateur	−2.49	−0.52	−2.60	84
Amateur	−1.63	−1.63	−3.42	82
Amateur	−2.20	−0.89	−2.88	83
Amateur	−1.70	1.13	0.02	94
Amateur	−2.72	−0.61	−2.83	82
Amateur	−2.76	−0.71	−2.98	82
Amateur	−1.13	2.10	2.01	86
Amateur	−2.71	−0.59	−2.81	82
Amateur	−1.28	−2.08	−3.76	80
Amateur	−2.72	−0.62	−2.84	82
Amateur	−1.54	−1.75	−3.51	81
Amateur	−2.68	−0.51	−2.69	83
Amateur	−1.91	−1.27	−3.16	83
Amateur	−2.85	−0.95	−3.30	80
			**Amateur mean ± SD**	**83 ± 4**

**Figure 5:**
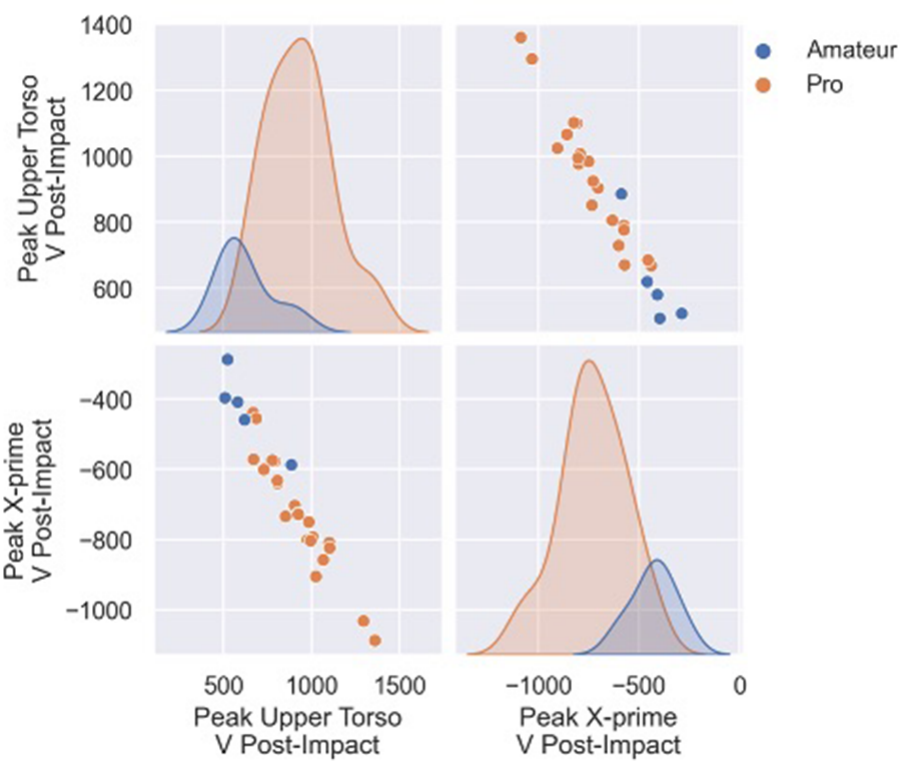
The relationship between peak upper torso rotational velocity post-impact and peak X-prime velocity post-impact, and their distribution in the pro and amateur groups.

Among the first 3 best performing models, peak X-prime velocity post-impact was also found to be a major contributor to the predictive model. Figure 5 illustrates the relationship between peak X-prime velocity post-impact and peak upper torso rotational velocity post-impact. This relationship, as well as the separation between professionals and amateurs, highlights the importance of follow-through as an indicator of performance.

## Discussion

Golf swing is a movement that generates power through the coordinated rotations of the pelvis and upper torso. Golfers have traditionally relied on handicaps to assess their skill, but the rating system takes many games to calculate and does not provide technical feedback for performance improvement. In addition, CSI and length of shot are commonly used to evaluate golf swings, but neither provide feedback on biomechanical swing technique nor provide feedback for proper rotational biomechanics to minimize injury. Here, we calculated parameters from nine variables narrowed to three rotational metrics to differentiate between pro and amateur swings. We included professional and amateur golfers with a wide variety of experience and training to capture a representative range of swings. The aim of this research is to propose a golf SPI to score golfers on swing technique based on the biomechanical parameters of the upper torso and pelvic rotation that correlate to performance outcomes. A golf SPI using the peak pelvic rotational velocity pre-impact, pelvic rotation at impact, and peak upper torso rotational velocity post-impact demonstrated the highest AUC when pro and amateur swings were classified using logistic regression with cross-validation (Figure 3).

We based our calculation of this index on methods established by prior gait indices in which a standardized population is compared to a separate population to quantify overall differences in parameters with a single outcome (20–22). We created a single-score index for the evaluation of golf swing rotational technique based on biomechanical metrics that had been found to be consistent in professional golf swings. In creating the golf SPI, we considered the inclusion of parameters thought to be technically relevant, reported in the literature, and those that demonstrated statistical significance in differentiating amateurs from pros in our data set. Previous research has shown that although downswing sequence and angular velocity peaks of the body segments may vary with swing style, the backswing and transition sequences are more consistent (5, 43). In addition, certain characteristics, such as a greater increase in the X-factor early in the downswing, are consistently found in pro golfers with high CSI (7). Capturing these defining characteristics can be accomplished by measuring joint angular velocities or joint torques (44). Here, we used 3D motion capture data to evaluate upper torso and pelvic rotational velocities, and to consolidate the defining characteristics of the pro golf swing into a single score, such that amateurs can readily quantify their swing technique as defined by key factors of the pro golf swing.

While this data was collected in a laboratory setting using 3D motion capture, these same metrics can be recorded using lightweight wearable inertial sensors, which currently measure rotational velocities with a low margin of error (45) and will soon offer an inexpensive, easy-to-use option for trainees. Wearable, lightweight inertial measurement unit (IMU) equipped devices open the door to real-time analytics on shoulder (upper torso) and hip (pelvic) rotation during on-the-field training. IMUs have been used with machine learning algorithms to identify specific segments of golf swing (17), recognize swing patterns that can lead to injuries (18), analyze clubhead speed (16), and provide feedback for wrist angles in uncocking motion (19). Integrating IMUs for motion analysis and summarizing the parameters that correlate to pro swing into a single score can assist with providing real-time feedback. This SPI demonstrated a strong association with pro vs. amateur golfers and is intended as an easily administered quantitative metric to augment coaching expertise.

### Limitations

In this initial setup, a limited sample size was used to create a continuous score for grading individual golf swings. Future research is needed to validate the SPI against a larger number of participants. Due to the limited number of participants, it is possible that the specific component structure, including the used kinematic metrics and the associated model parameters (i.e., PCA singular values) used to calculate the SPI, would be different in a larger sample. This research utilizes measurements obtained from 3D kinematic motion analysis; future research on rotational biomechanics of the golf swing from IMU-derived data is needed to validate and translate findings to the golf course and driving range.

### Implications

The SPI is an objective single-score index of golf swing rotational biomechanics representing golf swing performance compared to benchmark pros. The SPI, constructed from pro and amateur swings, may be a useful tool for detecting swing inefficiency and augmenting coaching, allowing for expedited learning and injury prevention.

## Data Availability

The raw data supporting the conclusions of this article will be made available by the authors, without undue reservation.
